# Alport Syndrome and Femtosecond Laser-assisted Cataract Surgery

**DOI:** 10.18502/jovr.v15i2.6748

**Published:** 2020-04-06

**Authors:** Paz Orts-Vila, Francisco Amparo, José Luís Rodríguez-Prats, Pedro Tañá-Rivero

**Affiliations:** ^1^ Ocular Motility Section, Oftalvist Alicante, Spain; ^2^ Cornea Service, Massachusetts Eye and Ear Infirmary, Department of Ophthalmology, Harvard Medical School, Boston, MA, USA; ^3^ Department of Clinical Sciences, Division of Health Sciences, University of Monterrey, Monterrey, Mexico; ^4^ Department of Cataract and Refractive Surgery, Clínica Vistahermosa, Oftalvist Alicante, Alicante, Spain

**Keywords:** Alport syndrome, Anterior Lenticonus, Cataract Surgery, Femtosecond Laser, Optiwave Refractive Analysis

## Abstract

We report the surgical management of a patient with bilateral anterior lenticonus due to Alport syndrome using femtosecond laser-assisted cataract surgery (FLACS) and the Optiwave Refractive Analysis (ORA) system. A 38-year-old man with Alport syndrome presented to our department with visual loss due to anterior lenticonus in both eyes. Adjustments during bilateral FLACS were performed with the software's calipers to manually delineate the anterior capsulotomy. Multifocal toric intraocular lenses (IOLs) were selected and placed in the posterior chamber with the aid of intraoperative aberrometry. The intended postoperative positioning parameters for the IOL as well as the planned visual acuity and refraction were achieved. The implementation of FLACS and intraoperative wavefront aberrometry is a safe and useful surgical approach for the management of cataract in challenging cases such as patients with anterior lenticonus due to Alport syndrome.

##  INTRODUCTION

Alport syndrome is a rare hereditary disease associated with renal failure and sensorineural deafness. Ocular anomalies are usually present in the X-linked or autosomal recessive forms of the disease.^[1]^ The most frequent ocular manifestations include anterior lenticonus, retinal abnormalities such as dot-and-fleck retinopathy and, less frequently, posterior polymorphous corneal dystrophy.^[2,3,4,5]^ Anterior lenticonus occurs in approximately 50% of men with X-linked Alport syndrome and can lead to rapid visual deterioration.^[6]^ Anterior lenticonus is a cone-shaped protrusion of the anterior lens capsule of the crystalline lens into the anterior chamber that leads to progressive lenticular myopia and astigmatism. Anterior lenticonus is absent at birth, but usually develops by the second or third decade of life^[7]^ and is bilateral in 75% of cases.^[8]^

Visual deficits due to anterior lenticonus can be managed successfully through the surgical removal of the crystalline lens.^[9,10,11,12]^ Phacoemulsification surgery has been shown to be an effective treatment for anterior lenticonus in the context of Alport syndrome; however, this treatment has two main pitfalls. The first pitfall is the intraocular lens (IOL) power selection and the correct positioning of the cylinder axis if a toric IOL is used, based on the corneal astigmatism. This is particularly important in special cases such as those with irregular astigmatism or a significant refractive contribution of the posterior corneal surface to the total corneal astigmatism.^[13,14]^ The second challenge is the high level of difficulty in performing anterior continuous curvilinear capsulorhexis (CCC) due to a structurally abnormal anterior capsule.^[4,15,16]^ The capsule's fragility can also cause problems when performing lens hydrodissection or the implantation of a foldable IOL.^[4,15,16]^ In addition, patients can develop spontaneous anterior capsule rupture prior to surgery.^[17,18]^ Femtosecond laser-assisted cataract surgery (FLACS) is a useful tool that helps to manage such challenges through automated, well-centered capsulotomy.^[19,20]^

In the current report, we present a case of bilateral anterior lenticonus secondary to Alport syndrome that was treated with FLACS and a toric IOL implantation whose power and orientation were verified using intraoperative aberrometry. Herein, we describe the surgical procedure and discuss some of the expected challenges associated with intraoperative imaging in such cases.

##  SURGICAL TECHNIQUE

A 38-year-old man who was previously diagnosed with Alport syndrome presented with bilateral progressive visual impairment over the previous five years. His medical history revealed sensorineural deafness and chronic renal failure requiring a kidney transplant 16 months prior to the presentation. The corrected decimal distance visual acuity (CDVA) was 0.8 in moderate photopic conditions in both eyes, but the patient experienced significant visual impairment under mesopic conditions. The patient described difficulty in performing daily tasks depending on light conditions, which had significantly increased in recent years. He was not able to tolerate even partial subjective refractive correction, which was –5.25 –7.25D x 180° OD and –6.75 –8.50D x 165° OS.

**Figure 1:**
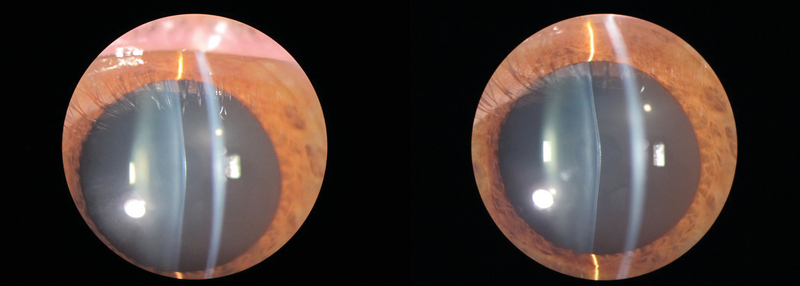
Slit-lamp view of the anterior lenticonus in the right and left eyes of the patient.

**Figure 2:**
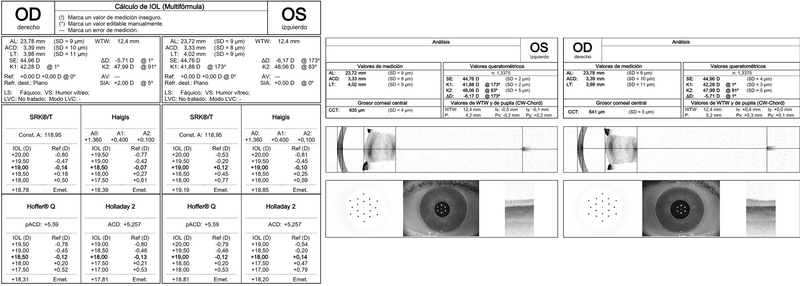
IOLMaster 700 report and a cross-sectional image of both eyes showing the anterior lenticonus projection into the anterior chamber.

**Figure 3:**
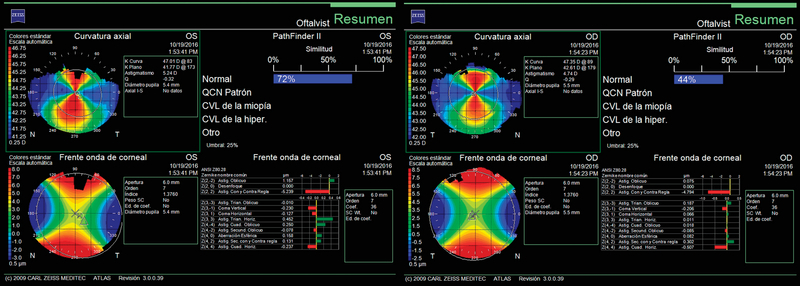
Atlas corneal topography (top) and Oculus Pentacam HD (bottom) maps of both eyes of the patient.

Anterior segment slit-lamp examination revealed bilateral anterior lenticonus [Figure 1]. Intraocular pressure was 15 mmHg OU, and the results of fundus examination in both eyes were normal. Biometric measurements obtained with an IOL Master 700 system (Carl Zeiss Meditec AG, Jena, Germany) showed an axial length of 23.78 mm OD and 23.72 mm OS and keratometry readings of 42.28 D x 1° and 47.99 D x 91° OD and 41.88 D x 173° and 48.06 D x 83° OS [Figure 2]. Corneal topography (ATLAS, Carl Zeiss Meditec AG, Jena, Germany) [Figure 3, top]. and tomography (Oculus Pentacam HD, Oculus Optikgeräte, Heidelberg, Germany) [Figure 3, bottom] showed regular astigmatism with enantiomorphism between the two eyes.

**Figure 4:**
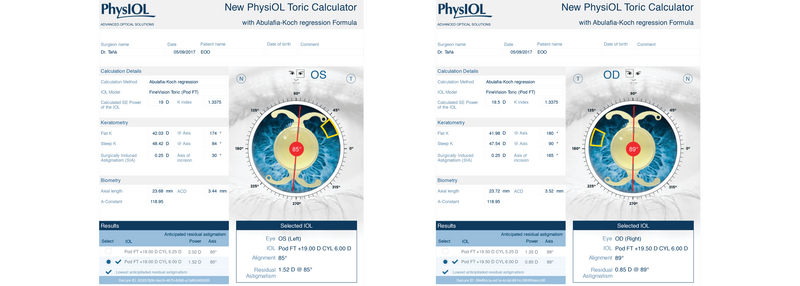
Proposed intraocular lens according to the PhysIOL toric online calculator.

IOL power was selected using the optical biometry (IOLMaster) and u online toric calculator (https://www.physioltoric.eu) with the Abulafia-Koch formula for the selected IOL (PhysIOL POD FT toric, Liege, Belgium). We consider it important to use a formula that takes into account the effect of the posterior surface of the cornea (such as the Abulafia-Koch formula used in this case) to achieve a preoperative plan that is as close as possible to the toroidal power suggested by the aberrometer. The predicted residual astigmatism was 1.49 D x 82° OD and 1.31 D x 84° OS [Figure 4], given that the highest available toric power for this type of IOL was chosen (6 D). Since an intraoperative aberrometer was available, we ensured that two additional IOL spherical powers (0.5 D above and below the determined power) were available in the operating room (there were no higher toroidal powers).

**Figure 5:**
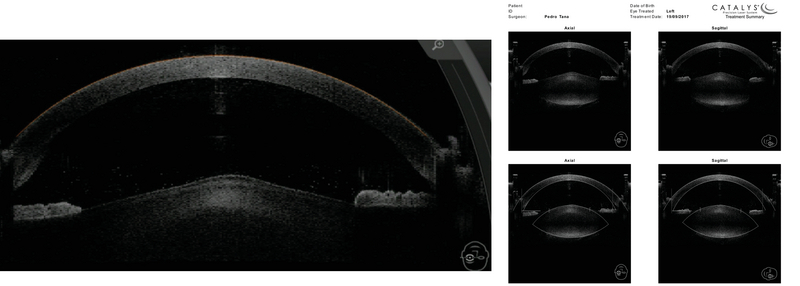
Intraoperative images confirming the presence of an anterior lenticonus in the anterior segment optical coherence tomography imaging interface.

Both eyes were operated under topical anesthesia with one-week interval between the two operations using a femtosecond laser system (Catalys, Johnson and Johnson, Cal, USA). The presence of an anterior lenticonus was confirmed through anterior segment optical coherence tomography (OCT) imaging captured intraoperatively using the Catalys interface [Figure 5]. The femtosecond laser was used to create a 5.0 mm capsulorhexis which was centered on the scanned crystalline cup. The standard removal of the lens nucleus and cortex was performed using phacoaspiration. IOL spherical and cylindrical power selection was refined under aphakic conditions using intraoperative aberrometry with Optiwave Refractive Analysis (ORA) (WaveTec Vision Systems, Inc., Alcon, Aliso Viejo, CA). The IOL power of both eyes was accurately matched with the preoperative calculations. Additionally, intraoperative aberrometry was used to verify the positioning of the toric IOL axis after the implantation of a capsular tension ring. Subtle pseudophakic rotations were performed, as guided by repeated aberrometer measurements, until the “NRR” (no rotation recommended) message was obtained.

Postoperatively, uncorrected decimal distance visual acuity improved to 0.9 in both eyes, and the CDVA improved to 1.0, with a refractive error of –0.50 D x 5° OD and +0.50 D –1 x 170° OS. Intermediate and near uncorrected decimal vision also improved to 0.9 and 1.0, respectively, in both eyes. Six weeks after the surgeries, both IOLs remained well centered.

The determination of the anterior capsule in the two eyes presented in this report was performed manually with precision, and the procedure was simple, being performed within a few seconds, and led to successful surgery in both eyes. Regarding the procedure, after the careful removal of the cohesive viscoelastic device, especially behind the IOL in the capsular bag, the IOL was placed along an approximate axis according to the preoperative calculations. The anterior chamber was then filled with balanced saline solution, and the edges of the incision were discretely hydrated, attempting to inject as little solution as possible while allowing spontaneous cohesion of the corneal incision edges.

##  DISCUSSION

Alport syndrome has a prevalence of 1/5000, and anterior lenticonus occurs in approximately 25% of these patients;^[3]^ this manifestation can lead to progressive lenticular myopia and irregular astigmatism. Anterior lenticonus is not present at birth and normally develops by the second decade of life; it is bilateral in 25% of patients and can lead to rapid visual deterioration.^[7]^ Clear lens extraction surgery with IOL implantation is known to be an effective option for visual rehabilitation in patients with anterior lenticonus. Compared to senile cataract, this procedure is more technically difficult in anterior lenticonus due to the fragility of the anterior capsule, which makes it susceptible to extension toward the lens periphery.^[21]^ The evidence shows that capsulotomy performed with FLACS shows better shape and size consistency than capsulotomy performed using conventional techniques.^[22,23,24,25]^ A well-shaped and centered circular 5 mm capsulorhexis is, in our experience, easier to achieve using the femtosecond laser. In cases where the OCT software cannot precisely identify the anterior capsule due to the lack of normal pattern recognition (e.g., lenticonus), its position can be easily defined manually using intraoperative real-time OCT.

The main difficulties that presented in the case described in this report were as follows: greater fragility of the anterior capsule; the calculation of the IOL's spherical and astigmatism power; and the determination of the cylinder axis for the toric IOLs to be implanted in the eyes with high corneal astigmatism.

Another dilemma in these cases is the selection of IOL, monofocal vs multifocal, especially when amblyopia has been ruled out. The selection should be made based on patient's needs and preferences as well as surgeon's discretion. If a multifocal IOL is selected, there is a possibility that an alternative IOL may need to be implanted in the case of surgical complications; thus, this scenario should be discussed with the patient in advance. The IOL model chosen in this report provides four points of support in the equator of the capsular bag, reducing unexpected rotation of IOL postoperatively.

The decision to use a toric trifocal IOL was based on the following: (i) the patient showed good previous visual acuity in photopic conditions, (ii) it is our preferred choice in procedures requiring a toric lens, (iii) the projected residual with-the-rule astigmatism was < 1D (potentially expected to decrease over time in young patients), and (iv) it was the highest torical power option available in the selected platform.

The topographic data obtained from Atlas and the Pentacam total astigmatism power were very similar. In fact, in similar cases (regular high with-the-rule or against-the-rule astigmatism), the incidence of astigmatism of the posterior surface of the cornea can be considered negligible, but not in the case of astigmatism of greater than 3D because of the proportionally large contribution of the posterior corneal astigmatism to the total corneal astigmatism.

At this point, it is generally difficult to determine the exact axis on which the IOL is actually positioned compared with the axis planned preoperatively since multiple subtle rotations are performed and we lack a transoperative method for reliably obtaining this information. However, the IOL implantation axis ultimately matched those planned by the online calculator in both eyes.

We routinely prefer the ORA system over any other manual or technological system in all our crystalline lens surgeries to both fine-tune spherical equivalents and determine the IOL's cylinder power and orientation. To determine the positioning of the toric IOL axis, we mark the 0 and 180° axes at the slit lamp to use it as a guide, but thicker marks reduce the accuracy. In our experience, even if matching marks are made with an Nd-YAG laser on the crystalline lens, the limbo-scleral and Nd-YAG marks are not aligned at the time of the surgery in many cases. Misalignments of 5–10° within the planned axis are frequent, with consequent residual astigmatism (approximately 1.5 D in an IOL with a cylindrical power of 6 D) as commonly observed. For this reason, we always fine-tune the position of toric IOLs using ORA, which has led to the significant improvement of our residual astigmatism outcomes.

In conclusion, the surgical and optical success of the approach for cataract surgery in Alport syndrome cases with anterior lenticonus presented herein highlights the importance of meticulous preoperative planning and surgical management in such challenging cases. Moreover, it highlights the critical aid that state-of-the-art technologies can provide to achieve the best clinical outcomes, especially with multifocal IOLs, due to the constrained margin of error that can be tolerated for their optimal performance.

##  Financial Support and Sponsorship

None.

##  Conflicts of Interest

There are no conflicts of interest.
